# Codon Usage Bias Correlates With Gene Length in Neurodegeneration Associated Genes

**DOI:** 10.3389/fnins.2022.895607

**Published:** 2022-07-04

**Authors:** Rekha Khandia, Mohd. Saeed, Ahmed M. Alharbi, Ghulam Md. Ashraf, Nigel H. Greig, Mohammad Amjad Kamal

**Affiliations:** ^1^Department of Biochemistry and Genetics, Barkatullah University, Bhopal, India; ^2^Department of Biology, College of Sciences, University of Hail, Hail, Saudi Arabia; ^3^Department of Clinical Laboratory Sciences, College of Applied Medical Sciences, University of Hail, Hail, Saudi Arabia; ^4^Pre-clinical Research Unit, King Fahd Medical Research Center, King Abdulaziz University, Jeddah, Saudi Arabia; ^5^Department of Medical Laboratory Sciences, Faculty of Applied Medical Sciences, King Abdulaziz University, Jeddah, Saudi Arabia; ^6^Drug Design and Development Section, Translational Gerontology Branch, Intramural Research Program National Institute on Aging, NIH, Baltimore, MD, United States; ^7^Institutes for Systems Genetics, Frontiers Science Center for Disease-Related Molecular Network, West China Hospital, Sichuan University, Chengdu, China; ^8^King Fahd Medical Research Center, King Abdulaziz University, Jeddah, Saudi Arabia; ^9^Department of Pharmacy, Faculty of Allied Health Sciences, Daffodil International University, Dhaka, Bangladesh; ^10^Enzymoics, Novel Global Community Educational Foundation, Hebersham, NSW, Australia

**Keywords:** gene length, compositional bias, codon usage bias, neurodegeneration, codon preference

## Abstract

Codon usage analysis is a crucial part of molecular characterization and is used to determine the factors affecting the evolution of a gene. The length of a gene is an important parameter that affects the characteristics of the gene, such as codon usage, compositional parameters, and sometimes, its functions. In the present study, we investigated the association of various parameters related to codon usage with the length of genes. Gene expression is affected by nucleotide disproportion. In sixty genes related to neurodegenerative disorders, the G nucleotide was the most abundant and the T nucleotide was the least. The nucleotide T exhibited a significant association with the length of the gene at both the overall compositional level and the first and second codon positions. Codon usage bias (CUB) of these genes was affected by pyrimidine and keto skews. Gene length was found to be significantly correlated with codon bias in neurodegeneration associated genes. In gene segments with lengths below 1,200 bp and above 2,400 bp, CUB was positively associated with length. Relative synonymous CUB, which is another measure of CUB, showed that codons TTA, GTT, GTC, TCA, GGT, and GGA exhibited a positive association with length, whereas codons GTA, AGC, CGT, CGA, and GGG showed a negative association. GC-ending codons were preferred over AT-ending codons. Overall analysis indicated that the association between CUB and length varies depending on the segment size; however, CUB of 1,200–2,000 bp gene segments appeared not affected by gene length. In synopsis, analysis suggests that length of the genes correlates with various imperative molecular signatures including A/T nucleotide disproportion and codon choices. In the present study we additionally evaluated various molecular features and their correlation with different indices of codon usage, like the Codon Adaptation Index (CAI) and Relative Dynonymous Codon Usage (RSCU) of codons. We also considered the impact of gene fragment size on different molecular features in genes related to neurodegeneration. This analysis will aid our understanding of and in potentially modulating gene expression in cases of defective gene functioning in clinical settings.

## Introduction

Several disorders, including neurodegenerative disorders, have been linked to age-related illnesses and dementias. Neurodegenerative disorders, of which there are multiple, relate to diseases instigated by dysfunction of neurons and pose a significant hazard to mental health (Gitler et al., 2017). They can severely impact a patient’s ability to move, speak, think, and even breathe (Przedborski et al., 2003). Ataxias (problems with mobility) and dementias are also common to neurodegeneration. Neurodegenerative disorders occur due to either inherited genetic changes, wherein defective genes are passed from one generation to the next (e.g., Huntington’s disease and familial Alzheimer’s disease) or a combination of hereditary and environmental factors (e.g., sporadic Parkinson’s disease which may potentially be caused by long-term exposure to toxic chemicals and/or pesticides, or an initiating factor such as head injury). However, aging is the most well-known factor contributing to neurodegenerative disorders (Research et al., 2017). Attempts are being conducted to halt or at least reduce the progression of neurodegeneration, and numerous natural and synthesized compounds as well as life-style changes are being evaluated in this context (Katsuno et al., 2012; Martier and Konstantinova, 2020; Sharifi-Rad et al., 2020; Bhattacharya et al., 2022; Onikanni et al., 2022). Dementia is described as cognitive and behavioral impairment involving “functional” impairments that significantly impact one’s daily activities (Josephs et al., 2009). Dysfunction and degeneration of brain cells and their interconnections can lead to irreversible dementia. Alzheimer’s disease (AD) dementia, vascular dementia (VaD), Lewy body dementia (LBD), and frontotemporal lobar dementia (FTD) are the four leading types of neurodegenerative dementias. The most well-known form of dementia, Alzheimer’s disease (AD), accounts for approximately 60% of all dementias, with the remaining 40% deriving from VaD, LBD, and FTD (Ripich and Horner, 2004). Major risk factors for dementia include hypertension, smoking, obesity, depression, physical inactivity, diabetes, minimal social interaction, hearing impairment, excessive alcohol consumption, traumatic brain injury, and air pollution (including high nitrogen oxide and carbon monoxide concentration) (Livingston et al., 2020). Interestingly, a genetic connection also has been found for dementia, and, in this context, next-generation sequencing has made large-scale molecular studies feasible. The genes associated with neurodegeneration need to be evaluated to decipher the molecular features of the subset of genes involved in dementias.

The role of various factors, such as selection, mutation, and composition in the evolution of a gene can be determined via studies on codon usage bias (CUB). CUB also provides insights into the differential architecture of a gene based on the gene’s function. Several synonymous mutations are involved in various ailments and underscore the fact that silent mutations can be detrimental. Furthermore, several studies have focused on various factors that affect CUB within and between species, such as the expression level, RNA stability, recombination rates, GC content, codon position, and gene length (Grishkevich and Yanai, 2014). Gene length is a particularly crucial parameter in the study of CUB that increases with time (partly owing to the insertion of transposable elements) and decreases with partial gene duplication. However, long gene length and high gene expression contribute equally to gene duplication and alternative splicing. In contrast, short gene length and low gene expression result in large gene families; thus pointing toward the origin of new trends in the genome (Behura and Severson, 2012). According to Urrutia and Hurst (2003), smaller gene length results in higher expression, smaller proteins, high amino acid bias, high codon bias, and less intronic material, which can evoke selective pressure to increase the efficiency of protein synthesis. The relevance of gene length to the early transcriptional responses of human fibroblasts toward serum stimulation has been studied, and the genome-wide transcriptional response was reported to be affected by gene length. Length indirectly regulates gene expression, i.e., shorter genes lead to faster protein production and subsequently participate in the control of longer proteins, which are produced later in the reaction (Kirkconnell et al., 2017). Many studies have suggested that gene length is involved in various biological processes that can ultimately lead to disorders such as cancer, neuronal dysfunction, and cardiomyopathies. Studies on different multigenic diseases show that gene length and splicing complexity are partially separated in defining cancer-linked pathways (Sahakyan and Balasubramanian, 2016).

Human genome studies on the functions of gene length, especially protein-coding genes, suggest that longer genes are expressed more in the brain, as well as in heart conditions and cancer. In contrast, smaller genes are involved mainly in the immune system and skin development; hence, genes with longer transcripts are predominantly associated with functions in the initial phase of development, whereas genes with shorter transcripts are crucial in everyday functions (Lopes et al., 2021). Accordingly, the compositional analysis of transcripts of AD shows that GC-ending codons are heavily skewed in the protein sequences (Yang et al., 2010), which suggests that the gene expression level plays a major role in the alteration of genes (Kalisz and Purugganan, 2004).

Gene length has been associated with gene expression in bacteria (Chiaromonte et al., 2003). In animals, short genes show strong expression, whereas, in plants, long genes show strong expression (Ren et al., 2007). Similarly, composition and gene length are correlated; short genes (<2,000 bp) show an increased percentage of GC3 than do long genes (Yang et al., 2021). A significant positive correlation has been reported between gene length and synonymous CUB in *Escherichia coli*, whereas a negative correlation has been reported in *Drosophila melanogaster* and *Saccharomyces cerevisiae*, which depicts the strong bias toward longer genes to enhance translational efficiency because of selection pressure (Moriyama and Powell, 1998). A significant correlation between the effective number of codons (ENC) and different skews (GC, AT, purine, pyrimidine, amino, and keto skews) has also been reported in *Wuchereria bancrofti* and *Schistosoma haematobium*, wherein all the skews were negative in both the organisms, except for that of pyrimidine in *S. haematobium*. This suggests a significant role of CUB on nucleotide disproportion (Mazumder et al., 2017); however, the different skews represent a marker for a specific genus and species.

The major purpose of this study was to analyze the characteristics that affect codon usage in neurodegenerative genes using various parameters such as relative synonymous codon usage (RSCU), ENC, nucleotide composition, and nucleotide skew calculations. An analysis of codon usage helps us better appreciate the role of gene length and evolutionary processes in determining codon usage of genes that may cause neurodegenerative diseases, thereby expanding our knowledgebase to improve current treatments and develop new therapeutic strategies.

## Materials and Methods

### Sequence Retrieval and Analysis

The genes responsible for neurodegeneration were obtained from the NCBI Genetic Testing Registry NGS Neurodegenerative disorders Multi-Gene Panel. A total of 183 transcripts belonging to 60 genes were downloaded and analyzed for the study. Both the start and stop codons were in the reading frame of the qualified genes and there were no ambiguous codons.

### Analysis of Composition and GC Content

The nucleotide components of the transcripts were analyzed. The overall percentage composition and percentage at the first, second, and third codon positions were also determined. Furthermore, the overall GC content and that at the three codon positions were determined. Compositional analysis was performed using the CAIcal software, as developed by Puigbò et al. (2008).

### Determination of Relative Synonymous Codon Usage

Not all 59 codons (excluding methionine and tryptophan encoded by a single codon and stop codons) are used equally to encode a protein. A bias in usage of the codon is depicted as an RSCU value, which denotes the relative frequency for which a codon is used, as compared to other codons of the family.

### Codon Adaptation Index

The Codon Adaptation Index (CAI) measures the similarity between codon usage and a reference set. It is a directional measure of codon bias and a predicting tool for the level of gene expression in an organism (Sharp and Li, 1987). The CAI values were calculated using the COUSIN tool, as developed by Bourret et al. (2019). The value of the CAI ranges between zero and one.

### Effective Number of Codons Determination

The ENC is a non-directional measure of codon bias (Song et al., 2017) with values ranging from 20 to 61. An ENC of 20 shows the highest bias, which means that only one codon of the codon family will be used for coding a single amino acid. An ENC of 61 shows the most negligible bias, indicating that all codons that are coding for a single amino acid will be used (Wang et al., 2018). The ENC values were calculated using the COUSIN tool developed by Bourret et al. (2019).

### Data Segregation Based on Length

The lengths of the transcripts ranged from 168 to 4,536 bp. Eight groups were prepared for a detailed investigation. The groups encompassed segments 1 (1–400 bp), 2 (400–800 bp), 3 (800–1,200 bp), 4 (1,200–1,600 bp), 5 (1,600–2,000 bp), 6 (2,000–2,400 bp), and 7 (2,400–4,500 bp). The eighth group contained full-length transcripts.

### Skew Calculations

Nucleotide skew indicates the bias in nucleotide usage. In a single strand of DNA, the frequencies of bases are not necessarily equal to those of its complementary strand; this difference often arises at the time of replication. For example, in leading strands, G and T are more abundant than C and A, which is attributed to mutational forces (Charneski et al., 2011). The skew is the normalized excess of one nucleotide over another in any given sequence (nucleotide A – nucleotide B/nucleotide A + nucleotide B). If the GC bias is zero, nucleotide A = nucleotide B.

### Scaled Chi-Square

Scaled chi-square (SCS) is another index for determining the codon bias, and it is a directional measure of CUB. Chi-square values indicate the deviation from the expected value; as the chi-square value highly depends on the length of the gene, the value is scaled by dividing the chi value by the number of codons present in a gene, excluding methionine and tryptophan, which do not contribute in chi. Therefore, using the SCS, genes of varying lengths can be compared (Shields and Sharp, 1987).

### Correlation Between Length and Codon Usage Bias

To investigate the effects of CUB on gene segments of various lengths, we divided our genes into different-sized segments. Eight groups were prepared: one group had complete gene lengths, whereas the other groups contained segments of 1–400, 400–800, 800–1,200, 1,200–1,600, 1,600–2,000, 2,000–2,400, and 2,400–4,500 bp.

### Statistical Analysis

Routine calculations such as addition and subtraction were performed in Microsoft Excel 2010. Statistical analysis, such as correlation analysis and principal component analysis (PCA), was performed using the Minitab 17 statistical software.

## Results

### Compositional Analysis

**FIGURE 1:**
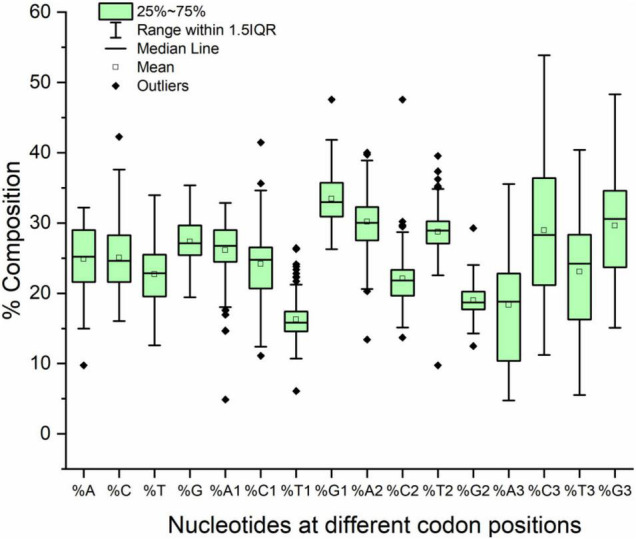
Box plot with outliers presenting nucleotide composition in neurodegeneration-related genes.

The compositional analysis showed that the overall mean GC content was higher than the AT content (52.41 and 47.58%, respectively). The same trend was observed in the composition at the first and third codon positions; however, at the second codon position, the AT2 content was greater than the GC2 content (58.94 and 41.05%, respectively). At the third codon position, a variance was observed for the A and G nucleotides (36.3 and 52.71%, respectively). The lowest variance was observed for T and G in the overall composition (13.82 and 9.74%, respectively) and at codon positions one (8.90 and 11.13%, respectively) and two (9.39 and 5.21%, respectively). Nucleotide composition at different codon positions is presented in Figure 1.

**FIGURE 2:**
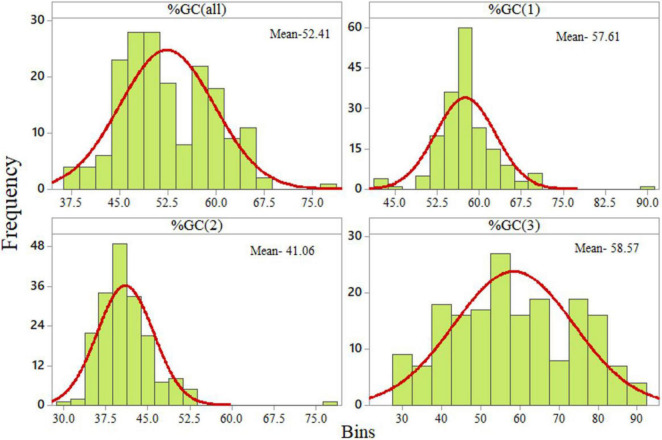
Distribution of GC content at various codon positions.

The GC content at the third codon position was widely distributed (from 27.73 to 89.60%). The distribution of the GC content at the second codon position was the lowest, ranging from 30.13 to 52.33% with one outlier at 76.82% (Figure 2).

### Correlation of Length With Composition

Correlation analysis is a statistical method used to determine the strength of a relationship between two variables. Such a relation could be either positive or negative. A negative correlation indicates that upon increasing one variable the second variable decreases and vice versa. In a positive correlation with an increase in one variable the other variable also increases. A higher correlation coefficient indicates a strong relationship, whereas a lower correlation indicates a weaker relationship. Correlation analysis was performed between the 20 compositional constraints (%A, %C, %T, %G, %GC, %A1, %C1, %T1, %G1, %GC1, %A2, %C2, %T2, %G2, %GC2, %A3, %C3, %T3, %G3, and %GC3) and the length of transcripts. The length was positively correlated with the overall nucleotide T component (T, *r* = 0.162, *p* < 0.05) and the T component at the first (T1, *r* = 0.147, *p* < 0.05) and second (T2, *r* = 0.258, *p* < 0.001) codon positions. A negative correlation was observed between C1 (*r* = –0.200, *p* < 0.01) and G2 (*r* = –0.198, *p* < 0.05).

### Correlation Between Length and Nucleotide Disproportion

All six nucleotide skews were subjected to correlation analysis with the length of genes. The length was negatively correlated with the AT skew (*r* = –0.204, *p* < 0.01). The nucleotide skews had no correlation with length.

### Correlation of Codon Usage Bias and Gene Expression With Skews

The correlation between various skews, gene expression level, and codon bias was evaluated, and it shows that SCS had a significant positive correlation only with the pyrimidine and keto skews. The CAI had a significant negative association with all skews except AT. This indicates that in the case of increased skew, protein expression is decreased; this association is independent of the AT skew.

### Correlation of Length With Codon Usage Bias

The SCS values were statistically positively associated with the complete length (*r* = 0.724, *p* < 0.0001) of genes associated with neurodegeneration. We investigated segments of various lengths for their association with CUB. In segments that had lengths below 1,200 bp and above 2,400 bp, a positive association was estimated (*r* = 0.940, *p* < 0.01; *r* = 0.638, *p* < 0.001; *r* = 0.332, *p* < 0.05, and *r* = 0.689, *p* < 0.001 for lengths 1–400, 400–800, 800–1,200, and 2,400–4,500 bp, respectively), whereas for segments ranging from 1,200 to 2,400 bp, CUB was not affected by length. The CUBs of the 400–800 and 2,000–2,400 bp segments were negatively correlated with each other (*r* =–0.448, *p* < 0.05).

### Correlation of Effective Number of Codons With GC3 Component

**FIGURE 3:**
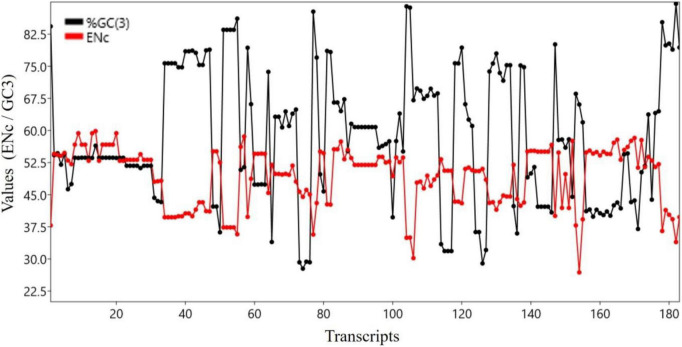
Trend analysis of ENC and GC3 components.

Both ENC and GC3 component are measures of bias. The ENC is a non-directional measure of CUB, with higher ENC values indicating lower bias (Hambuch and Parsch, 2005). GC3 is the frequency of G or C nucleotides at the third codon position and can be used to measure the CUB (Sahoo et al., 2019). There was a significant negative correlation between the ENC and GC3 (*r* = 0.683, *p* < 0.0001). Figure 3 shows the trends of GC3 and ENC for various transcripts.

### Effect of Length on Relative Synonymous Codon Usage Values

**FIGURE 4:**
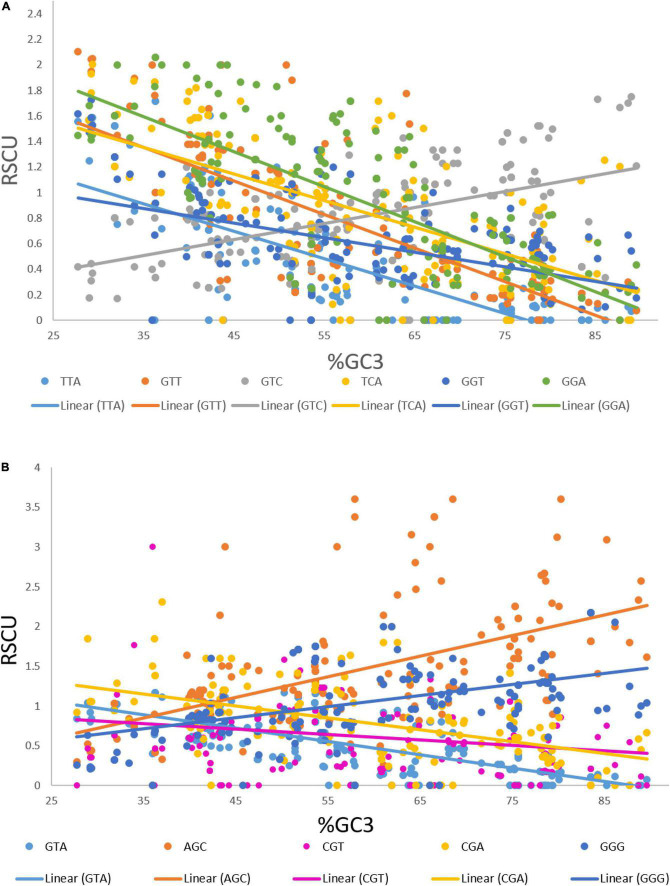
**(A)** Regression plot of GC3 and RSCU values of codons that were positively associated with gene length. **(B)** Regression plot of GC3 and RSCU values of codons that were negatively associated with gene length.

We performed a correlation analysis between the lengths and RSCU values of 183 transcripts. Eleven codons exhibited an association with the length of transcripts based on the RSCU values. Codons TTA (*r* = 0.181, *p* < 0.05), GTT (*r* = 0.194, *p* < 0.01), GTC (*r* = 0.18, *p* < 0.05), TCA (*r* = 0.225, *p* < 0.01), GGT (*r* = 0.182, *p* < 0.05), and GGA (*r* = 0.161, *p* < 0.05) showed a positive correlation with the length of the transcript (Figure 4A), whereas GTA (*r* = –0.236, *p* < 0.001), AGC (*r* = –0.252, *p* < 0.001), CGT (*r* = –0.200, *p* < 0.001), CGA (*r* = –0.153, *p* < 0.05), and GGG (*r* = –0.190, *p* < 0.01) showed a negative association with length. To investigate the correlation of these codons with the GC3 content, regression analysis was performed. The GC3 content was not correlated with length; however, an analysis was performed to investigate the effect of%GC3 on codons affected by length (Figures 4A,B). As shown in Table 1, among positively associated codons, GTT showed maximum variation (54.43%), whereas GGT showed minimum variation (23.73%); this was attributed to the GC3 content. GTA showed the maximum (44.38%) variation among the negatively associated codons, whereas CGT showed the minimum (2.16%) variation, which could be explained based on the GC3 content.

**TABLE 1:** Regression coefficients (GC3 vs. RSCU) of codons that were significantly associated with transcript length.

Codons positively associated with length	*R* ^2^	Codons negatively associated with length	*R* ^2^
TTA	0.5293	GTA	44.38
GTT	0.5443	AGC	31.12
GTC	0.2773	CGT	02.16
TCA	0.3825	CGA	25.39
GGT	0.2373	GGG	20.55
GGA	0.4778	–	–

### Effects of Different Lengths on Codon Usage Bias of Codons

Based on the RSCU values, we determined that TTA, GTT, GTC, TCA, GGT, and GGA exhibited a positive association with gene length, whereas GTA, AGC, CGT, CGA, and GGG showed a negative association. The manner via which CUB is affected over different ranges of transcript lengths was evaluated via regression analysis. Whether an association was constant along the length of the transcript or changed with it, was tested by generating eight groups based on different lengths, and then performing regression analysis. The eight groups encompassed segments of 1–400, 400–800, 800–1,200, 1,200–1,600, 100–2,000, 2,000–2,400, 2,400–4,500, and 1–4,500 bp.

### Negative Correlation of Length With Codon Usage Bias of Codons

**FIGURE 5:**
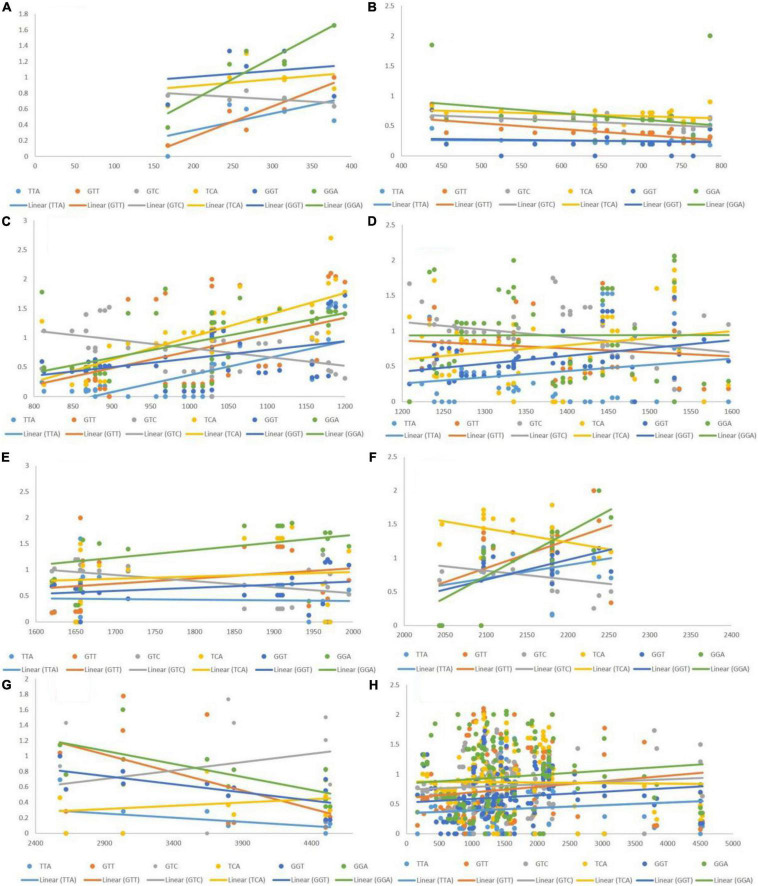
Regression analysis of RSCU values of codons (negatively associated with gene length) with gene segments of various lengths. Each dot represents a codon, the trend line is depicted as a solid line, and color coding for each codon and trend line is given inside the figure. **(A)** 1–400 bp; **(B)** 400–800 bp; **(C)** 800–1,200 bp; **(D)** 1,200–1,600 bp; **(E)** 1,600–2,000 bp; **(F)** 2,000–2,400 bp; **(G)** 2,400–4,500 bp; **(H)** 1–4,500 bp.

The post-regression r-squared values for each slope are presented in Table 2. At the whole transcript level, the RSCU values did not change considerably with the length of codons. However, in different ranges, a clear pattern was observed. Specifically, in the range 1–400 bp, the RSCU of the CGA codon was mostly affected by length (82.06%), followed by GTA (71.74%) and GGG (4.2%). In the ranges 400–800 and 1,200–2,000 bp, length did not affect the CUB of any codons. The overall analysis revealed that CGA experienced maximum variation in CUB, which was attributed to different length ranges. Figure 5 depicts the results, with the regression slopes.

**TABLE 2:** R-squared values (*R*^2^) ≥ 0.25 are shown in bold.

S. no.	Length	Negatively associated codons				
		GTA	AGC	CGT	CGA	GGG
1	0–4,400	0.0344	0.0561	0.0002	0.0105	0.0553
2	1–400	**0.7174**	0.0219	0.0376	**0.8206**	**0.472**
3	400–800	0.0137	0.2286	0.0135	0.1207	0.0566
4	800–1,200	0.1843	**0.5396**	0.0643	0.041	0.0811
5	1,200–1,600	0.1093	0.0107	0.0708	0.1714	0.0041
6	1,600–2,000	0.1669	0.0905	0.1246	0.0064	0.1544
7	2,000–2,400	0.1646	**0.3854**	0.1246	**0.4199**	0.0067
8	2,400–4,400	0.1804	Nil	0.0729	**0.4536**	0.0034

*The R^2^-value indicates the percentage of variation in the CUB of a codon that can be explained by length.*

### Positive Correlation of Length With Codon Usage Bias of Codons

**FIGURE 6:**
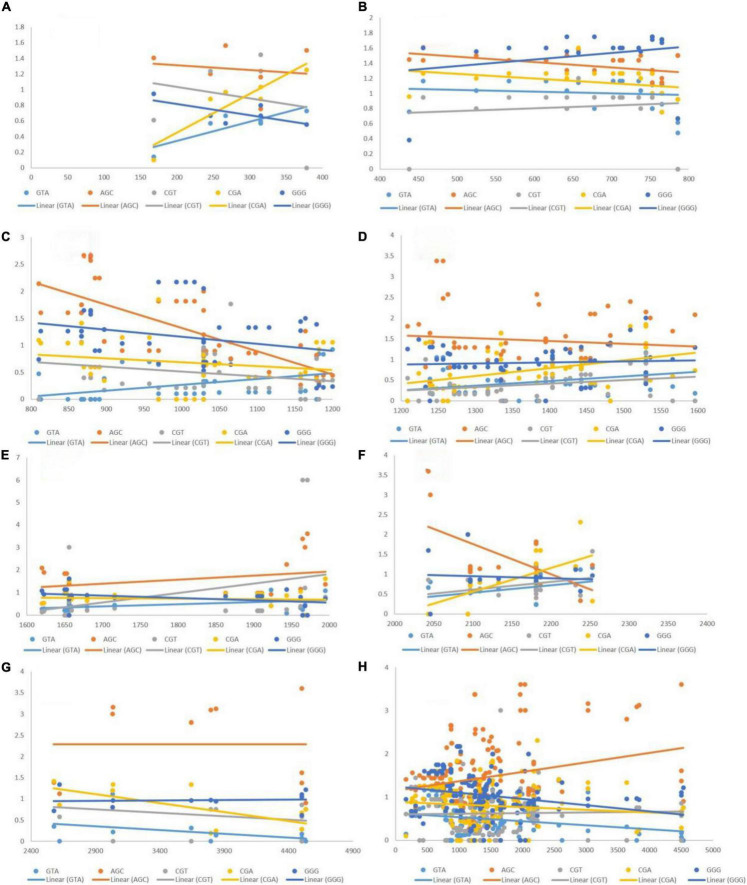
Regression analysis of RSCU values of codons (positively associated with gene length) with gene segments of various lengths. Each dot represents a codon. The trend line is depicted as a solid line, and color coding for each codon and trend line is given inside the figure. **(A)** 1–400 bp; **(B)** 400–800 bp; **(C)** 800–1,200 bp; **(D)** 1,200–1,600 bp; **(E)** 1,600–2,000 bp; **(F)** 2,000–2,400 bp; **(G)** 2,400–4,500 bp; **(H)** 1–4,500 bp.

The post-regression r-squared values for each slope are given in Table 3. At the whole transcript level, no considerable change in the RSCU values due to the length of codons was evident. The overall analysis of codons that were positively correlated to length revealed that GTT experienced the maximum variation in CUB (85.24%) followed by GGA (79.58%), across different length ranges. Similar to negatively linked codons, length did not affect the CUB of any positively correlated with codons in the ranges 400–800 and 1,200–2,000 bp. GGA experienced the maximum variation in CUB, which was attributed to the length of different ranges. Figure 6 depicts the results of the regression slopes.

**TABLE 3:** R-squared values (*R*^2^) ≥ 0.25 are shown in bold.

S. no.	Length	Positively associated codons					
		TTA	GTT	GTC	TCA	GGT	GGA
1	0–4,400	0.0059	0.0177	0.0083	0.0002	0.016	0.008
2	1–400	**0.338**	**0.8524**	**0.3967**	0.0778	0.0354	**0.7958**
3	400–800	0.0067	**0.513**	0.2169	0.0985	0.0092	0.0413
4	800–1,200	**0.5401**	0.2379	0.216	**0.5965**	0.1652	**0.3005**
5	1,200–1,600	0.0316	0.0166	0.1056	0.0442	0.1599	Nil
6	1,600–2,000	0.0017	0.0709	0.2279	0.0097	0.0856	0.0707
7	2,000–2,400	0.0684	0.2166	0.0985	0.2106	**0.3292**	**0.6082**
8	2,400–4,400	0.1741	**0.3166**	0.0673	0.0608	**0.4513**	**0.4911**

*The R^2^-value indicates the percentage of variation in the CUB of a codon that can be explained by length.*

### Principal Component Analysis

**FIGURE 7:**
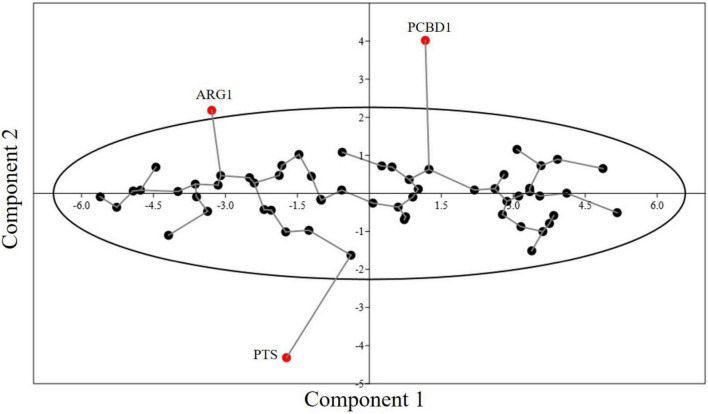
Minimum spanning tree graph for PCA of 60 genes involved in neurodegeneration. The eclipse shows a 90% concentration of genes within the area. Three genes are present outside the concentration eclipse, showing different behavior.

PCA is commonly used in dimensionality reduction. In Figure 7, axis 1 and axis 2 are plotted and the two axes account for most of the component. PCA is widely used to determine the major trend of codon usage. RSCU values of 59 synonymous codons are taken as a 59-dimensional vector. Methionine and tryptophan, which are encoded by single codons and three stop codons, are excluded from the study. PCA showed most genes to be scattered across the X-axis, and all genes (except *ARG1*, *PCBD1*, and *PTC*) were within the 90% confidence interval (Figure 7). There was not much variation in the codon usage of these genes. The first and second components contributed 51.42 and 6.07% of the variation, respectively. CUB was hence determined to be at a medium to low level.

### Genes With Special Feature

We analyzed the genes as to whether any display a feature different than present in other genes. For the same, we evaluated RSCU values of genes. Genes showing different behavior are presented in Table 4.

**TABLE 4:** The genes showing unusual behavior in terms of codon usage.

S. no.	Codons	Condition in other genes	% of genes showing common condition	Genes exhibiting contrary behavior
1	TTT	Underrepresentation	71.67	*MMADHC*
2	TCG	Underrepresentation	81.67	*ABCD1*
				*GCH1*
				*MMACHO*
3	GCG	Underrepresentation	71.67	*GAMT*
				*IVD*
				*MMAB*
4	CAA	Underrepresentation	75	*MAN2B1*
5	CGC	Underrepresentation	61.67	*DBT*
				*NPC2*
6	CAG	Overrepresentation	53.33	*MAN2B1*

The analysis revealed that there are 10 genes that reflect a different behavior than others. Here it is noteworthy that the codons TCG, GCG, and CGC are underrepresented in 81.67, 71.67, and 61.67% genes. This is an expected result since this codon contains the CpG dinucleotide, and CpG dinucleotide containing codons are found underrepresented in neurodegenerative disorders associated with brain iron accumulation (Alqahtani et al., 2021). CAG repeats are common in the *HTT* gene associated with Huntington’s disease (Guo et al., 2018) and overrepresentation of CAG may associate with it. Contrary to our result, the TTT codon encoding for phenylalanine is found overrepresented in cyclin genes. Underrepresentation of the CAA codon can potentially be understood by the fact that mutation in CAA might culminate into the TAA stop codon, and thus such underrepresentation might be to avoid premature termination of protein translation (DeRonde et al., 2022). Some genes exhibiting a codon usage pattern contradictory to maximum of the genes display their uniqueness and also might be associated with their physical and biological properties.

## Discussion

The frequencies of codon usage vary, which has been termed as codon bias. This is an imperative evolutionary phenomenon that operates from lower organisms to higher eukaryotes. Various hypotheses, such as selection-mutation equilibrium, genetic drift, and GC-biased gene conversion, have been presented to explain CUB in genes. In the present study, we evaluated the effect of transcript length on various parameters, such as codon bias, gene expression, and RSCU values of codons.

The nucleotide composition of any genome is an integral part of the molecular architecture of any gene. Composition is known to affect codon usage, as well as the choice and frequency of amino acids. An analysis of codon-pair repeats in 12 *Drosophila* genomes revealed the predominant usage of hydrophilic amino acids in NNG-CNN (a codon pair with G at the 3’ end of the first codon and C at the 5’ end of the second codon) (Behura and Severson, 2012). The importance of nucleotide composition was further underscored by Jørgensen et al. (2007), who analyzed the GC content in relation to bias and found a high association. Furthermore, genes located in GC-poor domains show higher deviation in bias and amino acid usage in *Apis mellifera*. In our analysis, G was most abundant, followed by A and C, with T being the least abundant. The GC3 content showed maximum variation and ranged between 30.26 and 87.75%, and it was crucial in compositional constraints. Wide variation in the GC3 content has been observed in freshwater turbellarians, cestodes, and trematodes, which could be easily separated into two distinct clusters based on the GC3 content range (Lamolle et al., 2019).

Estimation of a non-linear correlation between GC3 and a CUB measure revealed GC3 as a key factor in determining codon usage, and this application is independent of species (Wan et al., 2004). GC3 components of grasses resemble warm-blooded vertebrates, whereas those of dicot species resemble cold-blooded vertebrates (Montero et al., 1990).

In our study, ENC exhibited a negative association with GC3; in the case of higher GC3 content, a higher bias was seen (ENC is a non-directional measure of CUB). Our results are concordant with those of Huang et al. (2017), who also found a negative correlation (*r* = –0.243, *p* < 0.01) between ENC and GC3. Few studies have focused on the effects of the GC3 component on gene length (Duret and Mouchiroud, 1999; Wang and Hickey, 2007). In the present work, the GC3 component showed a correlation with the length of genes, although it was not significant. However, a negative correlation between GC3 and the length of the gene was found, suggesting that shorter genes have a higher GC3 content. Similar to our observation, gene length has been negatively associated with the GC3 component in *C. elegans*, *D. melanogaster*, *A. thaliana* (Duret and Mouchiroud, 1999), and *O. sativa* (Wang and Hickey, 2007). Reports have determined associations between gene expression level and length, with shorter genes showing strong expression levels in bacteria (Chiaromonte et al., 2003). A contrary trend has been observed in plants and animals, where longer genes in plants and shorter genes in animals are expressed more (Ren et al., 2007).

The codon composition is mainly affected by mutational and selective forces, including transcription, translation, mRNA stability, RNA and DNA methylation, co-translational folding, mRNA splicing, transport (Sharp and Li, 1986; Gebauer and Hentze, 2004; Bergman and Tuller, 2020), and genetic drift (Shah and Gilchrist, 2011), with CUB being affected by the codon composition (Bahiri-Elitzur and Tuller, 2021). When the effects of gene length on nucleotide composition were investigated, only the composition of T was found to be affected. Furthermore, the composition of T was affected by gene length at the first and second codon positions, while no nucleotide was correlated with gene length at the third codon position. The overall result indicated that the composition of T is imperative in deciding the gene length; in other words, gene lengths affect the composition of T in genes associated with neurodegeneration. Yang et al. (2021) linked a compositional parameter of the GC3 content to gene length, wherein genes shorter than 2,000 bp had a higher GC3 content than longer genes.

Significant amounts of the GC and AT skews can be explained based on the mutational differences between leading or lagging strands (Tillier and Collins, 2000). A comparison of the AT and GC skews in 30 avian mitochondrial genomes revealed that parrots have unusually strong compositional asymmetry (AT- and GC-skew) in their coding sequences (Eberhard and Wright, 2016). In *W. bancrofti* and *S. haematobium*, the GC, AT, purine, pyrimidine, amino, and keto skews were negatively associated with CUB, except for the pyrimidine skew in *S. haematobium* (Mazumder et al., 2017). Contrarily, in amphibians *Bombina bombina*, *B. fortinuptialis*, *B. lichuanensis*, *B. maxima*, *B. microdeladigitora*, *B. orientalis*, and *B. variegate*, no skew was correlated to CUB (Barbhuiya et al., 2019). The reports from other investigators suggest variable skewness in different genera and species, which could be a signature of the corresponding organisms. In the present study, the analysis of genes associated with neurodegeneration revealed the effect of CUB on the pyrimidine and keto skews.

In *S. cerevisiae*, *D. melanogaster*, *C. elegans*, and *A. thaliana*, a negative correlation between codon bias and gene length has been observed (Moriyama and Powell, 1998), while a positive correlation has been observed for *C. elegans*. In the present study, we investigated the effects of length on CUB in 60 genes and found no correlation. However, when we investigated various length ranges, a clear pattern of association between CUB and length was observed. In gene segments below 1,200 bp and above 2,400 bp, CUB was significantly positively associated with length. These results suggest that CUB operates in either small or large genes that are associated with neurodegeneration. Possibly, a selection pressure is acting principally on more minor genes, which are supposed to be highly expressed, and larger genes, which are energetically expensive. However, selection pressure cannot completely explain the correlation between length and codon bias. Additionally, the translational accuracy and speed model cannot explain the negative association between CUB and length in *S. cerevisiae*, *D. melanogaster*, *C. elegans*, and *A. thaliana* (Eyre-Walker, 1996; Duret and Mouchiroud, 1999). Marais and Duret (2001) reported that a negative correlation between codon bias and gene length is found only in eukaryotes, which contradicts our results, as we found a positive association of length and CUB in all cases.

When the correlation of the RSCU values of individual codons with length was investigated, only 11 out of 59 codons were found to be affected by the length of genes. Codons TTA, GTT, GTC, TCA, GGT, and GGA exhibited positive association, whereas codons GTA, AGC, CGT, CGA, and GGG showed a negative association with length. The GC3 percentage is an indicator of both compositional bias (Deka and Chakraborty, 2016) and codon bias (Shen et al., 2015). Therefore, we investigated the effect of the GC3 content on the CUB of these codons. Codons GTT and GTA (where G was at the first codon position and T at the second) were affected by the GC3 content, and 54.43 and 44.38% of the variation in the RSCU values of GTT and GTA, respectively, could be explained by the GC3 content.

No association between gene length and RSCU of vaccine-derived polioviruses, wild viruses, and live attenuated viruses was observed (Zhang et al., 2011). When the correlation between gene length and synonymous CUB was investigated for *D. melanogaster*, *E. coli*, and *S. cerevisiae*, a significant positive association was found in *E. coli* but negative correlations were found in *D. melanogaster* and *S. cerevisiae* genes. For *D. melanogaster* and *S. cerevisiae* genes, the ENC distribution, with respect to CUB, was different for short genes (300–500 bp) (Moriyama and Powell, 1998).

Our study found a significant positive association of SCS, a measure of CUB, with length, when the overall gene length was considered. Detailed investigation revealed that below 1,200 bp and above 2,400 bp, CUB had positive linkages with length, while in segments of 1,200–2,400 bp, there was no observed association, indicating that codon bias is present in both shorter and longer genes. However, in middle-length genes, CUB does not appear to occur. The results of the present study are concordant with those of a study by Moriyama and Powell (1998), wherein a higher bias was noted in energetically expensive longer genes, which occurred to maximize the translational efficiency, owing to selection pressure.

To further elucidate the effect of the length of various segments on RSCU, we performed regression analysis. Overall, the association of gene length with CUB was not evident. However, the RSCU values for both the GTT and GTA codons were notably affected by length (85.24 and 71.74%, respectively) in gene segments up to 400 bp. specifically, up to 400 bp, four out of six positively associated codons and three out of five negatively associated codons showed that the length was significantly correlated with RSCU values. In segments with lengths in the range 1,200–2,000 bp, CUB was not affected by length. Overall analysis indicated that the association between CUB and length varies depending on the segment size. The results were similar for both the overall CUB and CUB of specific codons.

Hia et al. (2019) demonstrated that human cells adopt a unique codon bias mechanism to modulate mRNA stability. Specifically, genes can be clustered in GC- and AT-ending codons; GC-ending codons enhance mRNA stability, while AT-ending codons destabilize mRNA. In the present study, GC-ending codons were noted to be preferred over AT-ending codons.

In contrast to that in other non-mammalian eukaryotes, CUB in humans is very high in both the highly and lowly expressed genes; selection possibly plays a role in both the enhancement and reduction of gene expression by promoting and hindering the use of optimal codons for the former and latter conditions. PCA based on the RSCU values revealed that the genes were not very scattered, and they were near the first axis. Overall, the genes exhibited a medium to low CUB.

## Conclusion

Codon usage analysis is an integral part of the molecular characterization of a gene. We studied how various compositional factors and other features, such as codon usage, might be affected by gene length. Compositional analysis revealed that G was the most abundant nucleotide, followed by A and C, with T being the least abundant. The distribution of the GC1 and GC3 contents was similar, with GC2 being the minimum. ENC was negatively associated with the GC3 content, which indicated that in genes with high GC3 content, the bias will be higher (ENC is a non-directional measure of CUB). Additionally, a negative correlation between the GC3 content and length indicated that longer genes had a lower GC3 content and lesser bias. In the present study, no effect of length on gene expression was observed. Of all four nucleotides, an association was found only between T and gene length; this effect was observed only at the first and second codon positions. Length affects the composition of gene nucleotides when T is considered. CUB in different organisms has been reported to be associated with different nucleotide skews; in this study, the pyrimidine and keto skews were found to be associated with CUB. To investigate the effect of gene length on CUB, we performed a correlation analysis, which showed no significant association between CUB and length at an overall level; however, when a segment-wise study was undertaken, a clear pattern was observed. CUB was statistically positively associated with length in gene segments below 1,200 bp and above 2,400 bp. This suggests that selection pressure is possibly acting on smaller genes, which are supposed to be highly expressed, and on larger genes, which are energetically expensive.

The RSCU values of eleven codons were significantly correlated with length. Codons TTA, GTT, GTC, TCA, GGT, and GGA exhibited a positive association with length, whereas codons GTA, AGC, CGT, CGA, and GGG showed a negative association. Analysis was performed to determine how the RSCU values of these codons are affected by the GC3 composition, and showed that codons GTT and GTA (containing G at the first codon position and T at the second) were affected the most by GC3. This revealed the positional significance of selective nucleotides and ultimately the role of selective forces balancing the RSCU values of codons and the composition and length of genes. The distribution of CUB is a documented variable for longer and shorter genes. In our study, CUB was affected by the length of genes when the gene length was shorter than 1,200 bp and longer than 2,400 bp. Among the codons that showed a significant association with the length of genes, GTT and GTA were maximally affected (85.24 and 71.74%, respectively). GC-ending codons were preferred over AT-ending codons, and PCA indicated that there was not much variation in the codon usage of genes.

Overall analysis indicated that gene length affects compositional bias and CUB (in the form of GC3 content). Gene length is correlated with the T content at the first and second codon positions, which are affected by selective forces, as well as the CUB of a few codons, with the maximal effect on codons that have G at the first position and T at the second. Gene length also affects the CUB of genes smaller than 1,200 bp and larger than 2,000 bp; the length of the gene significantly affects various parameters associated with codon usage.

## Data Availability Statement

The datasets presented in this study can be found in online repositories. The names of the repository/repositories and accession number(s) can be found in the article/supplementary material.

## Author Contributions

RK and MS: conceptualization. RK, MS, AMA, and GMA: methodology, software, analysis, writing, and editing. RK, NHG, and MAK: validation, writing-review and editing, and formal analysis. RK, MS, AMA, GMA, NHG, and MAK: funding acquisition. NHG: analysis/interpretation, writing/editing, approved, and manuscript accountability. All authors have read and agreed to the published version of the manuscript and read and agreed on the final version of the manuscript.

## Conflict of Interest

The authors declare that the research was conducted in the absence of any commercial or financial relationships that could be construed as a potential conflict of interest.

## Publisher’s Note

All claims expressed in this article are solely those of the authors and do not necessarily represent those of their affiliated organizations, or those of the publisher, the editors and the reviewers. Any product that may be evaluated in this article, or claim that may be made by its manufacturer, is not guaranteed or endorsed by the publisher.

## Abbreviations

CUB: Codon usage bias
A: adenine
T: Thymine
G: Guanine
C: Cytosine
RSCU: Relative synonymous codon usage
ENC: Effective number of codons
CAI: Codon Adaptation Index
SCS: Scaled chi-square
PCA: Principal component analysis.
